# Ultrasound-Guided, Mid-Forearm Median Nerve Block for Relief of Carpal Tunnel Syndrome Pain in the Emergency Department: A Case Report

**DOI:** 10.5811/cpcem.1265

**Published:** 2024-01-23

**Authors:** Daniel L. Puebla, Ilya Luchitsky, Roman Montes De Oca, Michael Shalaby, Robert A. Farrow

**Affiliations:** * Mount Sinai Medical Center, Department of Emergency Medicine, Miami, Florida; † Florida International University, Herbert Wertheim College of Medicine, Department of Emergency Medicine, Miami, Florida

**Keywords:** *case report*, *carpal tunnel syndrome*, *median nerve block*, *ultrasound guidance*, *regional anesthesia*

## Abstract

**Introduction:**

Carpal tunnel syndrome (CTS) is a common complaint in the emergency department (ED) and accounts for approximately 90% of all peripheral neuropathies.^6^ Pain control from injection with corticosteroids into the carpal tunnel space is associated with multiple possible complications including atrophy, iatrogenic median nerve injury, and skin changes. Ultrasound (US)-guided mid-forearm median nerve block is an ED procedure that can be used to avoid direct injection into the carpal tunnel space. Here we present a case report proposing the use of US-guided mid-forearm block as a safe and effective adjunct to the management of acute pain caused by CTS.

**Case Report:**

A previously healthy 44-year-old, right-hand dominant female presented to the ED with left wrist pain. Her clinical exam and US findings were consistent with CTS. Given her allergy to non-steroidal anti-inflammatory drugs, she was offered a median nerve block, which was performed in the ED. The patient reported continued pain relief 24 hours after discharge from the ED.

**Conclusion:**

There is limited data on the use of US-guided mid-forearm median nerve block as an acute pain management tool for CTS in the ED. The US-guided median nerve block done in the mid-forearm location can provide pain control for those with CTS while reducing the risk of complications associated with direct carpal tunnel injection.

Population Health Research Capsule What do we already know about this clinical entity?

*Carpal tunnel syndrome (CTS) presents with pain and paresthesias along the median nerve distribution. Treatment typically involves splinting and anti-inflammatories.*

What makes this presentation of disease reportable?

*We discuss a novel adjunct to pain control: ultrasound-guided mid-forearm median nerve block.*

What is the major learning point?

*This can be a useful adjunct for multimodal pain management in patients with CTS, avoiding complications associated with injecting into the carpal space.*

How might this improve emergency medicine practice?

*Mid-forearm median nerve block can improve pain management while reducing the incidence of CTS complications.*

## INTRODUCTION

Carpal tunnel syndrome (CTS) is a constellation of signs and symptoms that results from compression of the median nerve. It is the most common of all peripheral neuropathies (90% of cases) and is estimated to have a prevalence of 1–5%.^1^ ^,^ ^2^ Carpal tunnel syndrome is a clinical diagnosis, and the most common symptoms include numbness and tingling in the median nerve distribution, nocturnal numbness, weakness of the thenar musculature, positive Phalen test (sensitivity 42–85%, specificity 54–98%), and positive Tinel sign (sensitivity 38–100%, specificity 55–100%).^1^ ^,^ ^3^

An entrapment neuropathy caused by a combination of compression and traction, CTS causes changes in the microvascular structure of the nerve. This subsequently leads to increased permeability of the endoneurial vessels leading to edema of the median nerve.^4^ Ultrasound (US) may assist in the diagnosis of CTS by measuring the cross-sectional area of the median nerve. A median nerve cross-sectional area greater than 0.098 centimeters squared (cm^2^) with subjective findings on examination is 98% sensitive for the diagnosis of CTS.^5^

Direct injection of anesthetic and/or steroid into the carpal tunnel space can cause injury or weaken the median nerve.^6^ ^–^ ^8^ Studies show that injection nerve palsies have a reported incidence of 2% with median nerve palsies accounting for 3.6% of these complications.^9^ In this case report we propose the use of a mid-forearm, US-guided median nerve block as a safe and effective adjunct to the management of acute pain caused by CTS.

## CASE REPORT

**Image 1.**
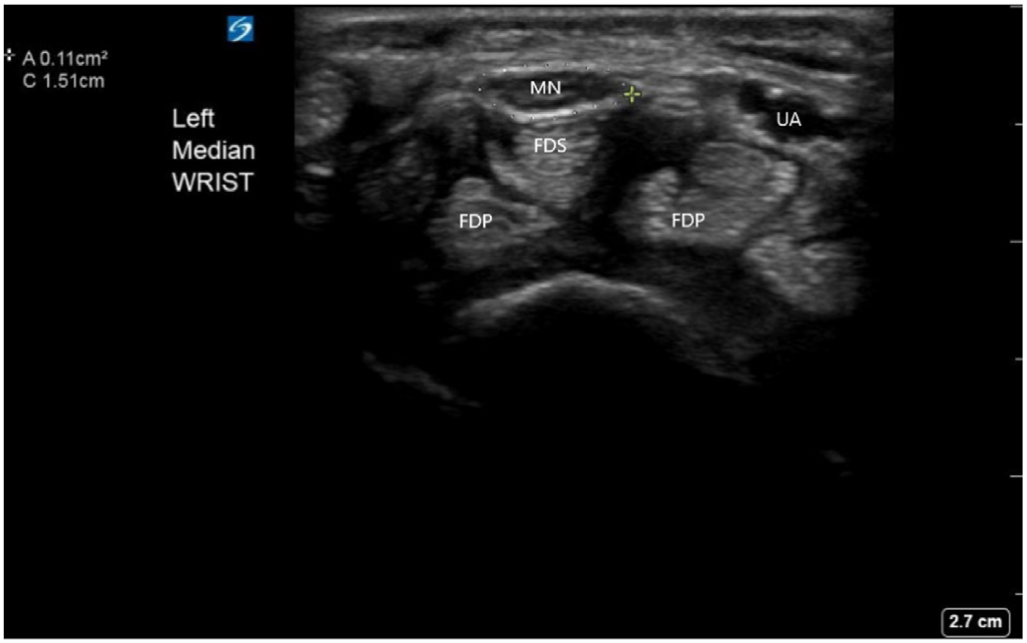
Carpal tunnel anatomy on ultrasound and median nerve (MN) sheath diameter. Surrounding the MN is the measurement tool indicating a MN sheath diameter of 0.11cm^2^, consistent with the diagnosis of carpal tunnel syndrome. *MN*, median nerve; *FDS*, flexor digitorum superficialis; *FDP*, flexor digitorum profundus; *UA*, ulnar artery.

A previously healthy, 44-year-old, right-hand dominant female presented to the ED with a chief complaint of two days of atraumatic left wrist pain exacerbated by wrist overuse secondary to her job as a waitress. Physical examination was notable for tenderness to palpation of the volar aspect of the left wrist and positive Tinel sign. Radiographs of the wrist were within normal limits. The median nerve was identified on US, and it demonstrated a cross-sectional area of 0.11cm^2^, suggesting the diagnosis of CTS (Image 1).

**Image 2.**
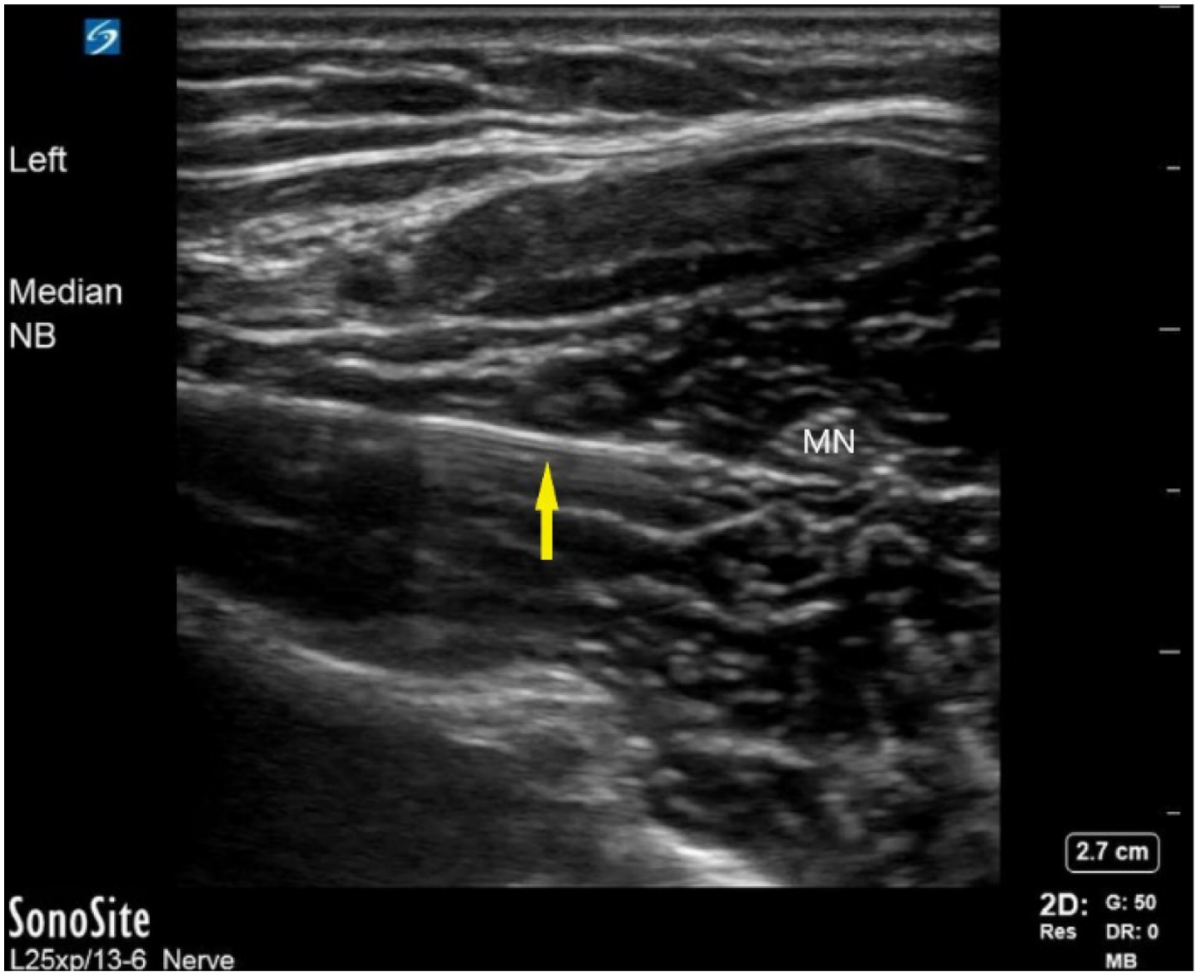
Median nerve block. This still frame of the median nerve block (NB) using a dynamic in-plane approach. The yellow arrow indicates the needle with underlying reverberation artifact. The needle is approaching from the left side of the screen along the fascia plane prior to injection of the anesthetic. *MN*, median nerve; *NB*, nerve block.

**Image 3.**
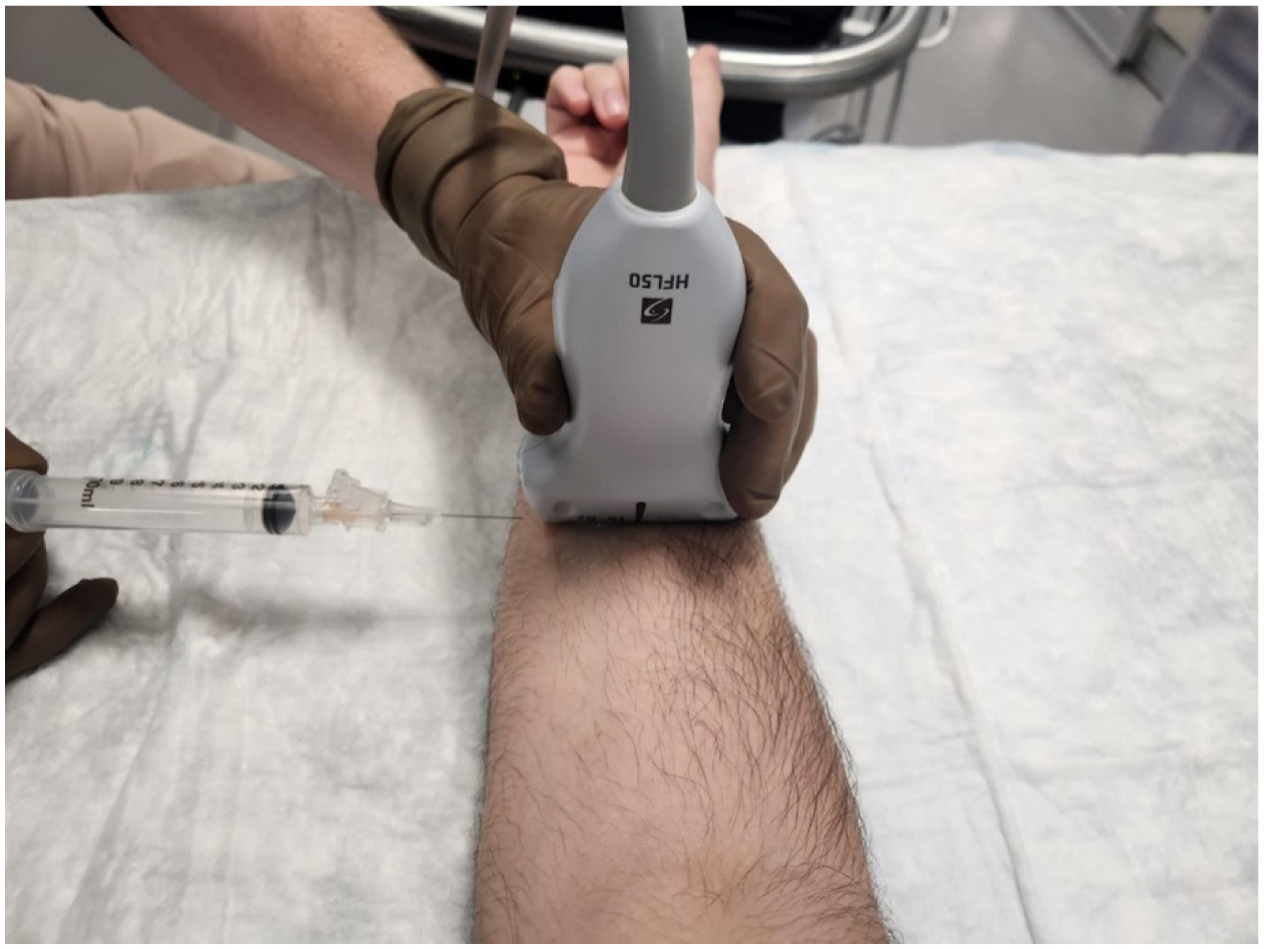
Representation of correct needle placement under ultrasound guidance using a linear high-frequency probe and needle. The in-plane lateral approach allows for complete needle visualization as the needle approaches the median nerve (MN). This image is used only for demonstration purposes of correct needle placement. The MN block should be performed under sterile conditions with a probe cover and a sterile field.

Pain management options were discussed with the patient. Since the patient was allergic to non-steroidal anti-inflammatory drugs (NSAID) and had minimal pain relief from acetaminophen, she was offered a median nerve block to which she consented. A pre-procedure US identified the median nerve between the deep and superficial flexor muscles, and a suitable location was selected to perform the nerve block. The mid-forearm median nerve block was performed using aseptic preparation and an in-plane, US-guided needle technique. A 27-gauge needle was used for the block with a 10-milliliter (mL) syringe filled with 10 mL of bupivacaine 0.5% without epinephrine. The fascial plane between the deep and superficial flexor groups was hydrodissected with bupivacaine until the median nerve was surrounded by anesthetic (Images 2 and 3).

The patient had significant pain relief within minutes. She was discharged in a Velcro volar splint and sling, with instructions to use acetaminophen as needed. At telephone follow-up 24 hours post procedure she reported significant improvement of her pain symptoms.

## DISCUSSION

Carpal tunnel syndrome management in the ED generally consists of NSAIDs in conjunction with splinting and follow up with a hand specialist. A study analyzing conservative management of CTS comprised of NSAIDs and night-time splinting demonstrated that approximately 25% of patients fail treatment with this approach.^10^ Another potential treatment is steroid injection; this requires injection directly into the carpal tunnel and ultimately offers short-term pain relief but no statistically significant long-term pain relief.^11^

The median nerve block is frequently performed in the ED as a means to effectively anesthetize the palmar aspect of the first three digits, the radial half of the fourth digit, and the distal dorsal portion of the second and third digits.^12^ The block is performed by extending the patient’s arm in the volar position, placing the probe in transverse position along the carpal tunnel space, and moving proximally until the median nerve is localized between the flexor digitorum superficialis and flexor digitorum profundus. The mid-forearm approach avoids direct injection into the carpal tunnel space, which can potentially cause median nerve injury.^2^ Using a sterile US probe cover during the procedure and appropriate sterile technique, approximately 5–10 mL of anesthetic can be safely injected for intended effect. Potential complications of this procedure include pain and discomfort during the injection, infection, and potential compromise of the brachial artery, which runs parallel to the median nerve.^12^ We recommend using the pre-procedure US to survey the selected needle path for any possible neurovascular structures.

There is limited data on the use of US-guided, mid-forearm median nerve block as an acute pain management tool for CTS in the ED. A study analyzing pain relief with forearm nerve block vs intravenous regional anesthesia (Bier block) in patients undergoing carpal tunnel release did show a statistically significant pain improvement at discharge with forearm block,^13^ demonstrating potential treatment viability. Steroids may be associated with complications such as increased numbness and tingling in hands in 5% of cases, transient sympathetic reaction in 2% of cases, skin depigmentation in 1.3–4% of cases, and atrophy in 1.5–40% of cases.^14^ Suggested benefits of using mid-forearm block include acute pain relief, avoidance of complications secondary to carpal tunnel space injection, and lack of complications associated with steroids.

## CONCLUSION

The case presented demonstrates the use of US-guided median nerve block as a potential adjunct treatment for pain management in carpal tunnel syndrome, which can be performed in the ED setting. The use of a median nerve block in a 44-year-old female with CTS provided significant pain relief, which was sustained at 24 hours follow-up. Future studies could evaluate the acute benefit of this treatment approach as an adjunct to NSAIDs, rest, and nightly splinting.
